# The Relation of Insanity to Civilisation.—II

**Published:** 1906-09-08

**Authors:** 


### THE RELATION OF INSANITY TO CIVILISATION.—II.

(*Continued from page* 371.)

### Madness in the Middle Ages.—Terrible Cures.

The dominant characteristics of the fifteenth and sixteenth centuries is a belief in magic and compacts with infernal spirits. Thus, new errors dethroned the old ones; but, as they were produced by the worst species of fanaticism, namely, religious fanaticism, they failed not to cause torrents of blood to flow. These were the times of magicians, of sorcerers, of persons possessed, and of demons and maniacs; these also were the times of exorcists, of inquisitors, and of thousands of unfortunate persons who expiated in torments and flames the misfortune of having lost their reason. It was about the year 1484 that magic began to play an important part in the affairs of Europe, and for two centuries and a half it strewed that quarter of the globe with its victims. Del Rio relates that 500 persons were executed at Geneva in 1515 during the space of three months. In Lorraine, in a period of sixteen years, Remigius caused 900 persons to be executed for sorcery, and has himself written an account of them. The registers of the Parliaments of Bordeaux, of Paris, and of Rome are filled with sentences of death for this crime (*Demonolatria ex judiciis.*) In Germany the progress of this moral contagion was no less remarkable. Hauber has prepared a list of the executions for sorcery that took place at Würzburg during two months and a half. More than 157 individuals were consigned to the flames in the course of 29 successive *autos-da-fé*. The greater portion of the individuals that share a place in this list are old women and strangers. It contains the names of children of twelve, of eleven, and even of nine years of age, of fourteen vicars of the cathedral, and of the most beautiful girl in the city. According to the best calculation it would appear that 100,000 persons were executed for sorcery in the German States during the rage of this delusion. This is placed beyond all doubt by the minutes of the legal proceedings of which they were the objects, from which it appears that they did not hesitate to avow their sexual connection with Satan, their nocturnal rendezvous with evil spirits, and to recognize the justice of the punishment inflicted on them. In those days of ignorance, the phenomena of nature, every marked excitement of the brain and the nerves, hysteria, epilepsy, chorea, and catalepsy, were all attributed to the agency of some malignant power. These illusions spread like contagious affections. In the celebrated affair of Moirag the malady first seized on children and then on women. The latter declared that they met the devil every night; that they rode on broomsticks and on spits; that when the devil was in a good humour he was in the habit of drawing the broomsticks from between their legs and beating them about the shoulders with it, bursting with laughter at the same time; and that they all had sexual connection with him. On August 25th, 1672, 62 persons, women and children, were condemned to death by the citizens of Dalicarlia, and burnt in the presence of many thousands of persons. These monstrous executions were endured for too long a time. In 1739 thirteen persons were consigned to the flames at Sigeden in Hungary; and it was not until 1749 that the series of these assassinations closed in Germany with the horrible process against Maria Renata of Würzburg.

### Victims of Scotch Superstition.

England did not remain free from the prevailing error of Europe; and, according to Barrington, 30,000 persons perished, the victims of these stupid accusations. But in no country was superstition so absurd, and at the same time so bloody, as in Scotland. It cannot for a moment be doubted that the Reformation produced madness in many minds. The fury with which Catholics opposed Protestants, the divisions in nations and families to which it necessarily gave rise, the inability of some men to comprehend the dispute, the want of the necessary educational preparation in others, and the zeal which animated all and drove them to bloodshed and battle, could not fail to disorder the intellect and disturb the reason. Luther himself could not escape the influence of his age. At one time he struggles with the devil, seizes him by the horns, and throws him to the earth; at another the devil gets the best of it, Luther shrinks from the combat, and escapes all bruised and beaten.

### Vampirism and Mental Maladies.

Vampirism, which, more than any other superstition, belongs to the history of moral medicine, and mental maladies prevailed at the commencement of the eighteenth century in many parts of Hungary, Bavaria, Silesia, and Lorraine. The peasants who were attacked with it believed that the souls of their enemies could not only appear to them, but commit acts of vengeance on them and their cattle, if the corpse did not get putrid or was not nailed down. Some imagined that these malignant spectres seized them by the throat, strangled them, and sucked their blood. Others fancied that they beheld them perpetrating these acts, and the malady became general. The terror inspired by such visions was so great that, after having endured it two or three times, the nerves of the patient were totally exhausted, and he died in a state of syncope. The evil was carried to such a pitch that the magistrates, in order to save the living, allowed them to violate the sanctuary of the dead. Such violations were carried on in the most formal manner. Citations were issued, witnesses heard, and the bodies most scrupulously examined; if the slightest sign of vampirism was discovered, the corpse was either burnt or nailed down by the hands of the common executioner.

### The Convulsionists.

It was about the same time that convulsionists made their appearance in France. The patients were seized with convulsions and cataleptic symptoms, rolled themselves on the ground, and tossed about their heads and limbs. Many of them were relieved by being soundly beaten. These accidents finished by degenerating into positive and manifest insanity. In all the fanatical sects of this genus the same distinctive features are to be traced—these, namely, an exalted and mystical devotion, hysterical phenomena, and an extreme state of nervous excitement leading directly to homicidal monomania.

### Revolution and Intellectual Disorders.

Political notions theretofore concentrated in a small circle of individuals were at this time about to spread among the masses. England, by the revolution of 1688, gave the signal for new disorders in the intelligence of man; her establishments were soon filled with insane persons; and it is remarkable that the chief victims were among the new nobility; whilst in France, when the hour of revolution did arrive, an inverse tendency manifested itself. The terrible cry of liberty that resounded from one end of France to the other did, indeed, agitate and overturn many minds. The ancient nobility were decimated by political reaction; and thus it happened that the establishments of Paris received a large number of titled individuals whom the overthrow of a dynasty that had endured for eight centuries, the wretched fate of their parents, and the destruction of their fortunes, had all combined to deprive of reason. Under the governments of the Republic and of Napoleon the organisation of the police spread terror and disquietude throughout France; and we light in consequence on a new form of mental alienation characterised by an excessive fear of being compromised, denounced, and arrested. The retreat from Moscow gave rise to a multiplicity of cases among both officers and soldiers, and the terror caused by the entry of the allied armies into France had a similar effect on the peasantry.

### French Madness.

The year 1815 was the epoch of political condemnation, of the exasperation of the warriors whose exploits had shed lustre on France, and also of a new species of monomania, of which profound misanthropy was the distinctive feature. The sudden descent from honours to obscurity, from wealth to poverty, the painful impressions made by the invasion of the soil, the disasters attendant on war, were all causes that could not but be powerful to unseat the reason of many. Many went stark mad for joy at the overthrow of the reigning dynasty; while, at a later period of time, the admissions were of persons whose intellectual faculties had been disturbed by disappointed ambition, ruined fortunes, and blighted affections. There was one great difference between the two revolutions; that of 1830 was effected in a short time, and therefore was less bloody, so that the number of persons whom it drove into madhouses is less than that of the victims of 1789 and the years that immediately succeeded.

### Mental Alienation in England.

From this general summary of the events, catastrophes, and revolutions that mark, with a particular impress, the aberrations of the human mind in each country we are naturally led to inquire into the particular cause of insanity in the different countries where this malady has been observed. The first remark that suggests itself on this topic is that the expression of madness varies according to the character of each people. By examining the nature of the causes of madness we shall discover the normal state of each nation. In England, where everything that is excessive in a great civilisation is to be found, mental alienation is very frequent. Many special causes contribute to this, such as ill-assorted marriages, which engender hereditary insanity, hazardous and desperate speculations, the frequency of commercial crises, the increasing fluctuations of political life, the laziness peculiar to the rich, the abuse of fermented liquors, and, lastly, the immense number of religious sects.

In France mental derangement is determined by vanity, ambition, pride, an immoderate love of pleasure, scepticism, and love. The sentiment of personality, as the French would call it, that is to say, inordinate self-love, and a certain inconstancy and fickleness of the ideas, were, at the time of the Gauls, as they are now, the distinctive characteristics of that great nation.

